# Progress in Balance Function and Vestibular Symptoms Following Vestibular Rehabilitation in a Patient With Left Cerebellar Infarction

**DOI:** 10.7759/cureus.83163

**Published:** 2025-04-29

**Authors:** Shingo Hirano, Tatsuya Igarashi, Hiroyuki Inooka, Tsubasa Mitsutake

**Affiliations:** 1 Rehabilitation, Saitama Yorii Hospital, Saitama, JPN; 2 Medical Science, Saga University Graduate School, Saga, JPN; 3 Physical Therapy, Faculty of Health Science Technology, Bunkyo Gakuin University, Saitama, JPN; 4 Clinical Research Center, Saga University Hospital, Saga, JPN

**Keywords:** balance impairment, cerebellar infarction, nystagmus, vertigo, vestibular rehabilitation

## Abstract

Vertigo and balance impairment are the primary symptoms of vestibular impairment. These symptoms affect an individual’s activities of daily living (ADL) and quality of life (QOL). In patients with stroke, vestibular symptoms vary depending on the area of damage, making it crucial to assess the appropriateness and effectiveness of vestibular rehabilitation (VR) according to each symptom and affected region. Here, we aimed to report the progress of balance function and vestibular symptoms after VR in a patient with left cerebellar infarction.

A 50-year-old male patient with left cerebellar infarction presented with mild trunk ataxia. Despite these mild impairments, the patient experienced vertigo and unsteadiness while standing and walking, necessitating assistance for fall prevention. The patient underwent VR for 28 days, including vestibulo-ocular reflex (VOR) cancellation exercises and habituation exercises. Subjective visual vertical (SVV), nystagmus, VOR cancellation test, grading of lateropulsion (GoL), Mini-Balance Evaluation Systems Test (Mini-BESTest), timed up and go test (TUG), modified Clinical Test of Sensory Interaction and Balance (mCTSIB) using a stabilometer, and self-reported vertigo intensity using a numeric rating scale (NRS) were measured biweekly.

Compared with the initial assessment, the patient’s performance on the SVV, VOR cancellation test, GoL, and TUG improved at the final assessment. Furthermore, on the Mini-BESTest, the score improved from 3/28 points to 26/28 points. All parameters of the mCTSIB using a stabilometer improved in the eyes-closed condition using foam rubber. However, left-beat nystagmus after head shaking, left-gaze nystagmus when gazing to the left, and vertigo during postural changes did not change.

Although VR in patients with left cerebellar infarction may improve balance function, its effectiveness against vertigo requires further investigation. These findings suggest that the mechanisms underlying the vestibular function in vertigo and balance are distinct. Future investigations are required to explore the effects of VR on lesions in the vestibular regions, considering the timing of the intervention, suitable cases, and appropriate frequency of sessions in greater detail.

## Introduction

Vertigo and balance impairment are the primary symptoms of vestibular impairment [1]. These symptoms affect activities of daily living (ADL) and quality of life (QOL) [2-4]. Vestibular function senses the rotational and linear acceleration of the head and contributes to the orientation of the head and the stability of eye movements in postural control. Moreover, vestibular function is related to maintaining balance while standing, walking speed, and the risk of falling [5-7]. Therefore, improving vestibular function is important for enhancing balance, decreasing the risk of falls, and improving QOL.

After input from peripheral organs, afferent vestibular information is transmitted to the vestibular nucleus located in the rostral medulla oblongata and caudal pons [8]. These signals are then relayed to the interstitial nucleus of Cajal in the midbrain before reaching the vestibular cortex, including the insular and parietal lobes, via the thalamus [9-11]. Therefore, if damage occurs at any site of the pathway, vertigo associated with vestibular impairment comes into existence.

Vestibular rehabilitation (VR) is a well-known form of rehabilitation used to improve vestibular impairment. VR is a rehabilitation program that aims to improve the vestibular and balance functions through three mechanisms: adaptation, substitution, and habituation. Vestibular and balance functions are optimized by VR in patients with unilateral peripheral vestibular impairments [12-15]. In recent years, the number of reports examining the effects of VR in patients with stroke has increased [16-18]. In patients with stroke, vestibular symptoms, such as the direction and magnitude of perceptual vertical deviation, presence or absence of vertigo, and eye torsion, vary, depending on the affected areas of the damaged afferent vestibular pathway [19]. For instance, cerebellar vertigo exhibits nystagmus and a direction of fall opposite to that observed in typical patients with unilateral peripheral vestibular impairments, often resulting in chronic symptoms [20,21]. Therefore, it is essential to assess the applicability and effectiveness of VR based on the specific symptoms and affected regions. However, there is a lack of reports detailing the longitudinal changes in vestibular and balance function following individualized VR in cerebellar infarction patients presenting with cerebellar vertigo.

This study aimed to report the progress of implementing VR, including balance training, in a patient with left cerebellar infarction who presented with rotational vertigo and impaired balance.

## Case presentation

**Figure 1:**
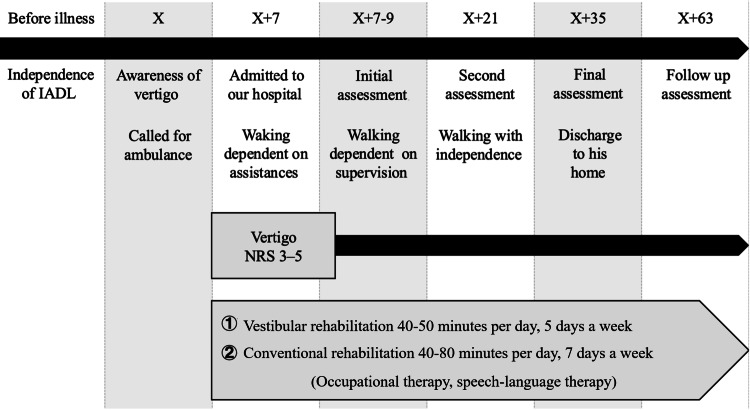
Patient’s disease progression This figure illustrates the progress of ADL independence and vertigo. 'X' represents the day the patient experienced a stroke and was transported to the acute hospital. Vestibular rehabilitation was conducted from day X+7 to X+35. Over time, the patient gradually regained mobility and was discharged home on day X+35. A follow-up evaluation was conducted on day X+63. IADL: instrumental activities of daily living; ADL: activities of daily living; NRS: numeric rating scale

Figure 1 illustrates the patient’s disease progression. The patient was a 50-year-old, right-handed male who called for an ambulance because of sudden subjective symptoms, including vertigo, nausea, and lightheadedness. Based on HINTS (short for head impulse-nystagmus-test of skew) examination, ataxia assessments (finger chase test, nose-finger test, and heel-shin test), and MRI findings, a diagnosis of cerebellar infarction was established, and the patient was admitted to an acute care hospital. The acute hospital treatment included pharmacotherapy with aspirin, ozagrel, and antihistamines, as well as rehabilitation; no surgical interventions, such as craniotomy, were undertaken. Following acute treatment, the patient was transferred to our hospital for rehabilitation on the seventh day post-ictus. He had no medical history of peripheral neuropathy, including diabetes or lumbar disc herniation. Moreover, the patient had no history of vertigo or dizziness.

On initial assessment, supine-to-sitting and maintenance of an upright stance were accomplished without supervision or assistance. Likewise, the sitting-to-standing did not require supervision or assistance, although the patient employed an external support to mitigate postural sway. However, the patient was aware of vertigo, with a severity of three to five on the numeric rating scale (NRS), with rapid head movements. The patient reported experiencing dizziness during ADLs, such as turning in bed, standing up, and observing the vortex of water in a flushing toilet. The patient complained of “being pulled to the right when walking, resulting in falls” and “experiencing vertigo when turning the head, making it impossible to remain standing.” Although the timed up and go test (TUG) was completed at 9.92 seconds and there was no change in time depending on the direction of the turn, the patient needed assistance for postural control in right-sided turns due to falling to the right. Similarly, the 10-m walking speed was 1.09 m/s, with no decrease in walking speed; however, the patient fell to the right and required assistance. The Mini-Balance Evaluation Systems Test (Mini-BESTest) scored 3/28 points; only the “sit to stand” and “stance, eyes open on firm and flat surface” items scored one and two points, respectively. Patients with vestibular impairments become unsteady and fall when standing or changing direction [5,6], which is consistent with the clinical findings. 

**Figure 2:**
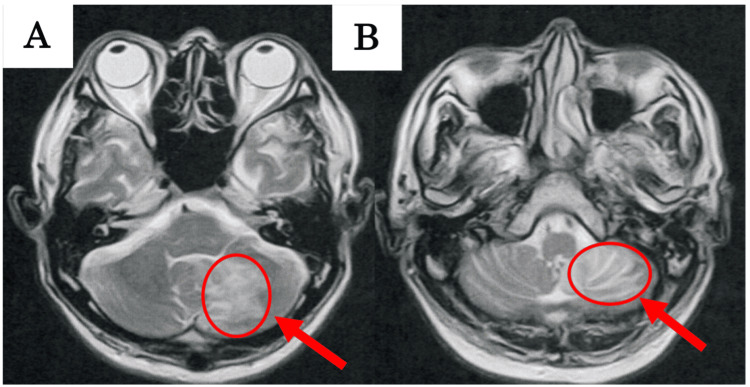
MRI at the initial assessment showing cerebellar infarction A: MRI image at the level of the middle cerebellar peduncle; B: MRI image at the level of the inferior cerebellar peduncle The arrow indicates the presence of an infarction in the flocculonodular lobe within the cerebellum.

Figure 2 shows the T2-weighted image of the patient at the time of initial assessment. A wide range of high-intensity signals were observed in the left cerebellar hemisphere and vermis at the level of the middle and inferior cerebellar peduncle [22]. Inhibitory fibers have been reported to reach the ipsilateral vestibular nucleus from the flocculonodular lobe via the inferior cerebellar peduncle [23], and it was speculated that the patient had similarly damaged fibers.

The subjective visual vertical (SVV) deviated 4.0 ± 1.2° to the right, and the head impulse test (HIT) did not show a catch-up saccade. Vestibulo-ocular reflex (VOR) cancellation test with visual confirmation was difficult on both sides. Spontaneous or positional nystagmus was not observed. Left-beat nystagmus was observed when shaking the head, and gaze nystagmus was noted when gazing to the left. The SVV measures the difference between the perceived and true vertical of the earth [24], and if the SVV deviates by >2.5°, vestibular impairment is estimated [24-26]. The SVV is a highly sensitive assessment tool for detecting peripheral and central vestibular impairments [27,28]. The HIT reflects the function of the lateral semicircular canal and is highly effective in distinguishing severe vestibular impairments [29]. In contrast, VOR cancellation expresses the function of inhibition fibers from the cerebellum to the lateral semicircular canal and assesses central vestibular impairments [30].

The results of the modified Clinical Test of Sensory Interaction and Balance (mCTSIB) using stabilometry are shown in Tables 1, 2. The mCTSIB is performed under four conditions: eyes open with and without foam rubber and eyes closed with and without foam rubber [31]. The mCTSIB reveals the interaction of each sensory modality during static standing and has high reliability and validity [31]. On initial assessment, the patient’s center of pressure (CoP) sway area increased in the eyes-closed condition, and the Romberg’s ratios of velocity were 1.93 cm/s and 2.63 cm/s in the without and with foam rubber conditions, respectively (Table 1). The Romberg’s ratio was the value obtained by dividing the velocity in the eyes-closed condition by that in the eyes-open condition. In the eyes-closed condition of the mCTSIB, vestibular function is more involved than in the eyes-open condition [31]. Additionally, we calculated the sensory weighting in static standing using the total path length of the CoP in the mCTSIB [32]. The visual, somatosensory, and vestibular ratios of the patient in this case report were 62.9%, 21.6%, and 15.5%, respectively, which exceeded two standard deviations from the visual ratio in healthy individuals [32]. Table 2 describes the results of the frequency analysis based on the Fast Fourier Transform (FFT) in the mCTSIB. The FFT is an algorithm that accelerates the discrete Fourier transform by converting time-domain functions into frequency-domain functions by decomposing discrete signals into a sum of trigonometric functions [33]. This method is often used to analyze the power spectra in stabilometry. On initial assessment, in conditions with eyes open with and without foam rubber, an increase in high-frequency oscillations (1.0-5.0 Hz) was observed in both the anteroposterior (AP) and medio-lateral (ML) directions. Under conditions with eyes closed with and without foam rubber, there was an increase in both high-frequency (1.0-5.0 Hz) and low-to-mid-frequency components (0.1-0.5 Hz) in both directions. Furthermore, the power across all frequency bands in both the AP and ML directions significantly exceeded the average values of age-matched healthy controls reported in previous studies [34] when tested under eyes-closed conditions. An increase in low-to-mid-frequency components (0.1-0.5 Hz) indicates vestibular dysfunction [34]. In patients with unilateral peripheral vestibular impairments, an increase in the power of low-to-mid-frequency (0.1-0.5 Hz) components is observed in both the AP and ML directions under eyes-closed conditions with and without foam rubber [35]. However, an increase in high-frequency components (1.0-5.0 Hz) may suggest a breakdown of the intermittent postural control model and adaptation to a continuous postural control model [36,37]. Similarly, in this patient, a trend consistent with these findings was observed, particularly under eyes-closed conditions, suggesting that the patient may have employed an abnormal postural control strategy due to unilateral vestibular impairment (Table 2).

**Table 1:** Progress of results of the stabiliometer test EO: eyes open; EC: eyes closed

Parameters		Initial assessment	Second assessment	Final assessment
Root mean square (cm)	EO without foam rubber	1.78	1.20	1.17
	EO with foam rubber	1.99	1.47	1.41
	EC without foam rubber	4.08	2.18	3.67
	EC with foam rubber	5.53	3.46	3.42
Velocity (cm/s)	EO without foam rubber	7.32	4.49	3.99
	EO with foam rubber	6.72	6.30	5.76
	EC without foam rubber	14.15	9.44	17.62
	EC with foam rubber	17.66	13.47	13.40
Romberg’s ratio	Without foam rubber	1.93	2.10	4.41
	With foam rubber	2.63	2.14	2.33
Ratio of sensory (%)	Visual	62.94	56.45	36.22
	Somatosensory	21.62	30.90	57.54
	Vestibular	15.44	12.65	6.25

**Table 2:** Progress of results of the frequency analysis AP: anterior-posterior direction; ML: medio-lateral direction; EO: eyes-open; EC: eyes-closed; LF: low frequency (0.01–0.1 Hz); L-MF: low-mid-frequency (0.1–0.5 Hz); M-HF: mid-high-frequency (0.5–1.0 Hz); HF: high frequency (0.1–5.0 Hz)

Parameters		AP			ML		
		Initial assessment	Second assessment	Final assessment	Initial assessment	Second assessment	Final assessment
Total power of LF components (cm)	EO without foam rubber	0.40	0.16	0.21	0.38	0.07	0.39
	EO with foam rubber	0.60	0.13	0.29	0.57	0.40	0.29
	EC without foam rubber	0.76	0.40	1.01	0.76	1.06	1.87
	EC with foam rubber	1.02	0.65	0.76	2.24	0.60	1.21
Total power of L-MF components (cm)	EO without foam rubber	2.02	0.66	1.43	2.87	1.02	2.20
	EO with foam rubber	3.00	1.64	1.91	3.51	2.82	1.83
	EC without foam rubber	5.37	2.30	4.55	11.18	5.47	6.06
	EC with foam rubber	8.29	7.48	5.47	11.29	8.21	6.36
Total power of M-HF components (cm)	EO without foam rubber	3.65	2.45	1.67	4.11	2.17	2.14
	EO with foam rubber	1.98	2.34	1.92	4.60	3.17	2.82
	EC without foam rubber	4.64	3.67	6.00	3.81	3.27	7.21
	EC with foam rubber	5.90	3.97	4.12	6.21	3.90	4.60
Total power of HF components (cm)	EO without foam rubber	4.19	2.69	1.88	4.29	2.11	2.62
	EO with foam rubber	3.78	3.55	3.86	5.28	4.01	3.62
	EC without foam rubber	10.73	6.19	13.27	7.62	5.88	10.46
	EC with foam rubber	12.03	9.24	9.86	11.47	7.55	7.98

Grading of lateropulsion (GoL) was classified as grade III, indicating that the patient fell and required assistance to maintain posture when standing with the eyes closed. Contralesional body and head tilts were observed while standing and walking; however, no skew deviation or ocular movement disorders were noted. Because measuring ocular torsion requires special equipment, it is difficult to perform this assessment. The patient’s tilt direction was opposite to that of patients with Wallenberg syndrome, who often presented with a tilt toward the ipsilesional side [38].

Additional findings included a Mini-Mental State Examination (MMSE) score of 30/30 points, with no significant impairment in higher cognitive function. The Fugl-Meyer Assessment (FMA) scores were perfect: 34/34 points for lower extremity motor function and 24/24 points for sensory function. The Trunk Impairment Scale (TIS) also showed a maximum score of 23/23, indicating no motor paralysis, sensory deficits, or trunk function impairment. The Scale for the Assessment and Rating of Ataxia (SARA) resulted in a score of six, with four points for gait and two points for stance, indicating no upper or lower limb ataxia. The trunk ataxia assessment was at stage II, suggesting mild trunk ataxia. Ataxic gait characteristics, such as reduced walking speed and instability in multiple directions, have been reported [39]. However, the patient showed no reduction in walking speed. Although lateral instability to the right was observed, no AP instability was observed. Thus, trunk ataxia alone cannot fully account for the instability observed in the patient. Cerebellar vertigo in the patient was primarily attributed to the disinhibition of the vestibular nuclei on the damaged side caused by damage to the inhibitory fibers from the unilateral flocculonodular lobe to the corresponding vestibular nuclei [20], as suggested by the lesion site, episodes of instability, vestibular function tests, and stabilometry assessments.

This case report was published after obtaining written informed consent from the patient following a thorough explanation of the treatment methods and confidentiality protection, both verbally and in writing. Additionally, the guidelines of the Consensus-based Advisory Report Explanation statement were referenced in this case report [40].

Intervention protocol

**Figure 3:**
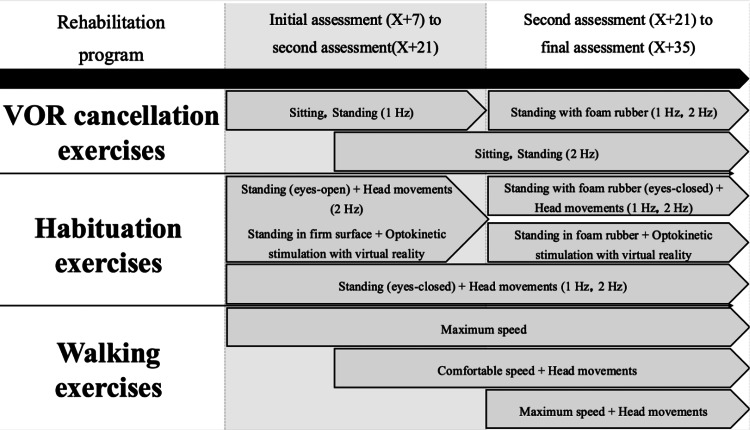
Progress of vestibular rehabilitation This figure illustrates the changes in difficulty level during vestibular rehabilitation over time. 'X' represents the day the patient experienced a stroke and was transported to the acute hospital. To prevent learning effects, attempts were made to modify the difficulty of tasks as time progressed and as balance function improved. The difficulty was adjusted by changing posture (sitting, standing, standing on foam rubber) and increasing the speed of head movements. VOR: vestibulo-ocular reflex

Previous studies have reported that VR, including habituation exercises, improves balance function and walking ability and alleviates vertigo in patients with stroke, including those with cerebellar lesions [16,41]. As previously mentioned, VR involves adaptation, substitution, and habituation. Adaptation results from tissue remodeling due to the upregulation of genes and proteins in response to specific sensory information, whereas habituation is achieved by reducing the excitability of postsynaptic potentials through the inhibition of presynaptic calcium channels [42]. Substitution refers to the central plasticity that occurs through neural activity induced by sensory conflicts [43]. Considering the mechanism of cerebellar vertigo and these reports, VR, particularly through habituation exercises, may contribute to reducing the excitability of postsynaptic potentials and correcting activity in the vestibular nuclei, thus potentially improving vestibular function in the patient. Therefore, VR was implemented to enhance balance function and walking ability and to reduce vertigo. Figure 3 illustrates the VR sessions implemented at various stages for the patient. The VR program, aimed at improving vestibular and balance function, consisted of VOR and habituation exercises, which were started at the time of the initial assessment.

**Figure 4:**
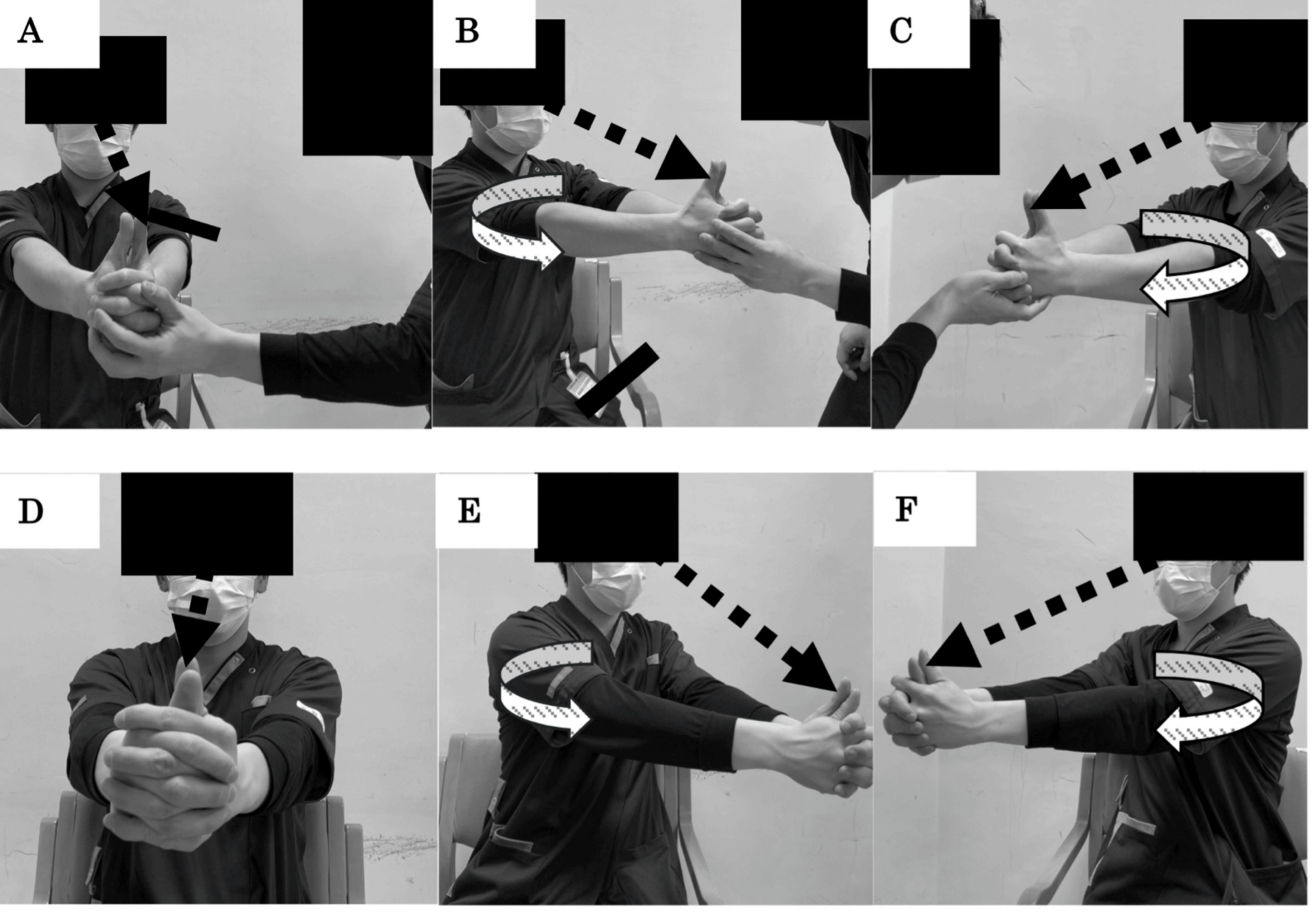
VOR cancellation exercises A: Starting position of the VOR cancellation exercises with passive movement; B: Left rotation of the VOR cancellation exercises with passive movement; C: Right rotation of the VOR cancellation exercises with passive movement; D: Starting position of the VOR cancellation exercises with active movement; E: Left rotation of the VOR cancellation exercises with active movement; F: Right rotation of the VOR cancellation exercises with active movement Throughout the exercises, the patient maintains fixation on his thumb while performing head and trunk movements. The difficulty level can be adjusted by changing postures (sitting, standing, standing on foam rubber) and varying the speed of the head movements. VOR: vestibulo-ocular reflex

VOR cancellation exercises (Figure 4) are used in VR to induce adaptation for the rehabilitation of central vestibular dysfunction. These exercises enhance the control mechanisms of the cerebellum and cerebral cortex over the vestibular nuclei [44]. This program involved simultaneously rotating the head, trunk, and upper limbs while fixating on the tip of the thumb. The VR sessions began with the patient in the seated position. The patient’s self-perceived vertigo and instability were monitored, and once these symptoms subsided, the difficulty progressively increased to standing and then to standing on foam. The rotational speed was set to the maximum level that the patient could tolerate without inducing vertigo during the exercise. The exercises were performed for two minutes per set, totaling 10 minutes over five sets, with appropriate rest intervals in between. Vertigo induced after VOR cancellation was rated between one and three on the NRS and subsided within approximately one minute.

Habituation exercises, which also serve as balance exercises, were performed while standing or walking and included head rotations, lateral flexions, and AP bends, which provoked vertigo and instability. No fixed gaze on the target was maintained during head movements. For standing, exercises commenced with eyes-open conditions without foam, progressing to more difficult conditions such as eyes open with foam and eyes closed with and without foam, as the subjective feeling of instability subsided. Walking exercises included not only head movements but also direction changes that frequently caused instability and vertigo in the patient. Additionally, in response to the patient’s reports of vertigo when seeing swirling water in the toilet, VR under visual motion stimulation was conducted using virtual reality goggles (ELECOM, VRG-D02PBK; ELECOM Co., Ltd., Osaka, Japan) attached to a smartphone. The SVV application (Kuroda ENT Clinic, Yatsushiro, Japan) was used to randomly set the speed within a range of 15-45°/s. Standing exercises were performed for either 12 or 24 minutes, incorporating two sets of each head movement direction with breaks as needed, matching the typical rehabilitation session duration. Visual motion stimuli and walking exercises were performed for 10-20 minutes per regular rehabilitation session. These VR sessions were conducted for 40-50 minutes per day, five days a week, with physician approval. In addition to VR, occupational therapy (ADL and fine motor skills exercises) and speech-language therapy (oral function and craniofacial muscle exercises) were performed for approximately 40-80 minutes per day, seven days a week.

Assessments and analysis

Assessments were conducted at the time of admission to our hospital and again at 14 and 28 days post-admission.

The vestibular function tests included the SVV, VOR cancellation test, nystagmus, recent episodes of vertigo within the last week and their contexts, and self-reported intensity of vertigo using an NRS ranging from 0 (none) to 10 (very strong). The SVV was performed using virtual reality goggles (ELECOM, VRG-D02PBK) equipped with a smartphone, a specific remote control (ELECOM, JC-XR05BK, ELECOM Co., Ltd.), and a neck brace (Dr. MED, DR-127, Duk-In Co., Ltd., Seoul, South Korea). The patient performed the test in a seated position, wearing a neck brace and virtual reality goggles, and operated the SVV application (Kuroda ENT Clinic) downloaded to the smartphone via remote control. Each measurement was randomly conducted on the left and right sides 10 times, with the starting angles randomly set between ± 30° and ± 70°. The mean and standard deviation of the 10 trials were recorded. Clockwise tilts were recorded as positive (+), and counterclockwise tilts were recorded as negative (-). For the VOR cancellation test (Figure 4), the examiner passively rotated the patient’s head, trunk, and upper limbs while instructing the patient verbally to keep his gaze fixed on the tip of his thumb. The patient’s eye movements during and immediately after rotation were observed to determine whether there was a delayed saccade toward the thumb, indicating difficulty with the VOR cancellation test. A nystagmus test was performed because the patient did not exhibit spontaneous nystagmus; hence, head-shaking and gaze-evoked nystagmus were assessed. Head-shaking nystagmus was performed in a dark room in a seated position, rotating the patient’s head approximately 10° at approximately 2.8 Hz horizontally for 15 seconds with eyes closed and then observing any nystagmus upon opening the eyes [21]. Gaze-evoked nystagmus was assessed under similar conditions by having the patient’s gaze at a target positioned approximately 40° to either side for approximately 10 seconds [45].

Balance function was assessed using the GoL [38] and mCTSIB. The mCTSIB was evaluated using a force plate (BW-6000; sampling frequency, 100 Hz, Anima Technologies Pvt. Ltd., Bengaluru, India) and a specific foam rubber (natural rubber; tensile strength, 2.1 kgf/cm²; density, 0.06 g/cm²; thickness, 3.5 cm, Anima Technologies Pvt. Ltd.,). The measurement posture was in a quiet standing position with the heels touching and toes out at 30° [31]. The mCTSIB involved four conditions, as already mentioned, each performed once for 30 s, and the CoP was measured. Subsequent analyses were used to calculate the root mean square (RMS) and velocity for each condition. Although previous studies recommended three trials per condition [46], only one trial per condition was conducted owing to time constraints within routine clinical settings and participant fatigue. A single trial in patients with stroke has been reported to exhibit high reliability and validity [47]. Additionally, as previously described, the visual, somatosensory, and vestibular sensory ratios were calculated while the patient was standing, using the total path length from the mCTSIB. The sensory scores of visual, somatosensory, and vestibular were calculated. Visual score (Vis) was calculated according to the following formula [32]: LX eyes-open/LX eyes-open with foam rubber. Somatosensory score (SOM) was determined according to the following formula [32]: LX eyes-open/LX eyes-closed. Vestibular score (Ves) was calculated according to the following formula [32]: LX eyes-open/LX eyes-closed with foam rubber. Here, LX represents the total path length from the mCTSIB. 

Subsequently, these sensory scores were converted into percentages as the sensory ratios. The ratio of visual was calculated by the following formula [32]: Vis/(SOM + Vis + Ves) × 100. Similarly, the ratio of somatosensory was determined by the following formula [32]: SOM/(SOM + Vis + Ves) × 100. The ratio of vestibular was also calculated by the following formula [32]: Ves/(SOM + Vis + Ves) × 100. Furthermore, frequency analysis using FFT was performed, and the power in low-frequency (0.01-0.1 Hz), low-to-mid-frequency (0.1-0.5 Hz), mid-to-high-frequency (0.5-1.0 Hz), and high-frequency (1.0-5.0 Hz) components was calculated for each condition in both the AP and ML directions.

Walking assessments included 10-m walking speed, TUG, functional ambulation category (FAC), and Mini-BESTest. Both the 10-m walking speed and TUG reflect walking ability and dynamic balance in patients with stroke, with high reliability and validity [48,49]. The Mini-BESTest, consisting of 14 items with a total of 28 points, indicates a higher balance function with higher scores and better reflects dynamic balance with less ceiling effect and higher reliability and validity than the BBS [50,51]. These walking tests are recommended as core outcome sets for patients with stroke [52]. The functional independence measure (FIM) was used to evaluate ADL independence, and the NRS was used to assess patient-reported outcomes related to instability during ADL, including difficulty and frequency of unassisted standing, walking, and turning. Follow-up assessments were conducted 56 days after admission (28 days post-discharge) to evaluate fall history and the Life-Space Assessment (LSA), a self-reported measure quantifying the range of life activities, with a maximum score of 120 indicating a higher range of activity [53]. All the assessments were performed by a single physical therapist. 

Results

Tables 1-3 present the assessment results at each time point. The SVV changed from 4.0 ± 1.2° at the initial assessment to -0.1 ± 1.8° at the final assessment, and improvements were noted in the VOR cancellation test. The GoL score changed from grade III at the initial assessment to grade I at the final assessment, indicating the resolution of falls while standing with the eyes closed. However, rotational vertigo was observed throughout the VR period, and the self-perceived intensity of vertigo remained at three to five on the NRS. No changes were noted in head-shaking nystagmus or gaze-evoked nystagmus.

**Table 3:** Comparison outcomes between each assessment points SVV: subjective visual vertical; HIT: head impulse test; VOR cancellation test: vestibulo-ocular reflex cancellation test; NRS: numeric rating scale; GoL: grading of lateropulsion; Mini-BESTest: Mini Balance Evaluation Systems Test; TUG: timed up and go test; FAC: functional ambulation category; ADL: activities of daily living; FIM: Functional Independence Measure; SARA: Scale for the Assessment and Rating of Ataxia

Parameters	Initial assessment	Second assessment	Final assessment
Days after onset (days)	7	21	35
SVV (mean ± SD°)	4.0 ± 1.2	0.3 ± 1.3	-0.1 ± 1.8
HIT	Normal	Normal	Normal
VOR cancellation test	Abnormal	Abnormal	Abnormal
NRS of vertigo (points)	5	5	5
Head-shaking nystagmus	Left-beat nystagmus	Left-beat nystagmus	Left-beat nystagmus
Gaze-evoked nystagmus	Left-gaze nystagmus	Left-gaze nystagmus	Left-gaze nystagmus
Head tilt (direction)	Right	Right	Right
GoL	Grade III	Grade I	Grade I
Mini-BESTest (points)	3	25	26
10-m walking speed (m/s)	1.09	1.12	1.47
TUG (s)	9.92	7.47	7.32
FAC	2	3	5
FIM (points)	87	101	121
NRS of instability in ADL (points)	7	3	2
SARA (points)	6	2	2

In the mCTSIB, most parameters decreased in the eyes-closed condition with foam rubber (Table 1). Table 2 presents the results of frequency analysis at each time point. At the final assessment, a decrease in high-frequency components was noted, particularly in the eyes-open condition with and without foam rubber and the eyes-closed condition with foam rubber, compared with the initial assessment. Furthermore, in eyes closed with and without foam rubber, a notable decrease in the low-to-mid-frequency components was observed. In the ML direction, a decrease in low-to-mid-frequency components was noted in the eyes-open condition with and without foam rubber conditions at the final assessment. In the eyes-closed condition with and without foam rubber conditions, a decrease in low-to-mid-frequency components was observed, and in the eyes-closed condition with foam rubber, reductions in both the low- and high-frequency components were noted. Additionally, the initial weighting ratios of the visual, somatosensory, and vestibular contributions were 62.9%, 21.6%, and 15.5%, respectively; however, at the final assessment, they changed to 36.2%, 57.5%, and 6.3%, respectively, falling within ± 2 standard deviations of the normal range for healthy individuals [32].

Between the initial assessment and the final assessment, the 10-m walking speed improved from 1.09 m/s to 1.47 m/s, the TUG score improved from 9.92 s to 7.32 s, and FAC improved from 3 to 5. Additionally, the tendency to fall to the right during walking resolved, and no assistance was required, including during directional changes. The Mini-BESTest score improved from 3/28 points at the initial assessment to 26/28 points at the final assessment. In the Mini-BESTest, the score for the single-leg stance and standing on foam was 1 point at the final assessment. The FIM score improved from 87/126 at initial assessment to 121/126 at final assessment. Additionally, the NRS score for instability in daily living improved from seven to two points at the final assessment. No falls were reported during the follow-up assessment, and the LSA score was 75/120 points. No adverse events or significant worsening of symptoms were observed during the VR implementation period or after rehabilitation.

## Discussion

The patient had no motor paralysis or sensory function decline, and motor ataxia was mild. Therefore, it was assumed that these factors had little effect on instability during walking. Lesions and instability, along with vestibular function and stabilometric tests, suggested that disinhibition of the unilateral vestibular nuclei and excessive reliance on visual inputs affected the patient’s instability and rotational vertigo during movement. Previous studies have reported that VR, including habituation exercises, improves balance function and walking ability and alleviates vertigo in patients with stroke, including those with cerebellar lesions [16, 41]. Considering the mechanism of cerebellar vertigo, VR, particularly focusing on habituation exercises, could contribute to reducing the excitability of postsynaptic potentials and correcting activity in the vestibular nuclei, potentially improving vestibular function. Thus, VR was implemented to enhance balance function and walking ability and alleviate vertigo in the patient.

VR is a rehabilitation approach aimed at improving the vestibular and balance functions using three mechanisms: adaptation, habituation, and substitution. In the patient in this case report, guided by recommendations in the guidelines [54], VOR cancellation and habituation exercises specifically targeted adaptation and habituation, which are considered effective in improving cerebellar vertigo. Approximately 30 days of VR led to improvements in static and dynamic balance, walking speed, and ADL-related instability. Furthermore, within approximately 14 days, the patient achieved a change of >3 points on the Mini-BESTest at each evaluation, which is the minimal important change (MIC) [55]. The patient also experienced improvements in walking speed, TUG, and FIM. A previous study has shown that patients with infratentorial stroke, including cerebellar infarcts, conducted combined balance training incorporating VR exercises, such as GSE and VOR cancellation, daily for 20~30 minutes for more than 42 days [41]. Significant improvements in balance and walking ability were observed, with improvements exceeding the MIC for the BBS [41]. Although the patient aligned with the age and VR initiation period of the participants in Balci et al.’s study [41], previous studies have not specifically examined patients with cerebellar infarction. This case report suggests that VR during the subacute phase can contribute to improvements in the balance and walking ability of patients with cerebellar stroke. 

Two potential mechanisms can be considered for the improvement in balance function observed in the patient through VR. The first possibility is the correction of asymmetry in the vestibular function. Previous studies have reported that improvements in lower limb motor paralysis [56], sensation [57], trunk function [58], and cognitive function [59] are associated with enhanced independence in ADL and balance function. However, in the patient, no changes were observed in these functions, but improvements in ADL independence and balance were noted, along with improvements in the VOR cancellation test and SVV. As mentioned earlier, cerebellar vestibular disorders primarily involve disinhibition of the vestibular nuclei on the ipsilateral side due to injury to the inhibitory fibers from the ipsilateral flocculonodular lobe [20]. Moreover, habituation reduces synaptic post-potential excitability by inhibiting presynaptic calcium channels [42]. Furthermore, VOR cancellation exercises promote control mechanisms in the vestibular nuclei via the cerebellum and cerebral cortex [44]. Thus, VR focused on habituation exercises, and VOR cancellation exercises might have corrected the imbalance in excitability between the bilateral vestibular nuclei by suppressing excessive excitability on one side, contributing to an improvement in balance function.

The second possibility is that, rather than an improvement in vestibular function, sensory re-weighting in postural control contributed to an improvement in balance function. Sensory re-weighting is the process by which the brain changes the reliance on sensory input during postural control and movement based on the environment and its own capabilities [60]. Although improvements were observed in SVV, the VOR cancellation test, and balance function, no changes in vertigo or nystagmus were noted. Additionally, at the initial assessment, a visually dominant postural control strategy was evident. However, at the final evaluation, it shifted to a somatosensory-dominant strategy. Excessive reliance on visual inputs in patients with vestibular impairments is associated with increased fall risk and decreased balance function [61]. Internal models of the head and body, such as the SVV, are modulated by somatosensory inputs from the neck, lower limbs, and trunk, and reflect vestibular compensation [62, 63]. Therefore, sensory reweighting in postural control contributes to the improvement of balance function in the patient. Although it is challenging to support these hypotheses conclusively, this is the first case report documenting the course of VR in a patient with cerebellar infarction-induced vestibular dysfunction and proposing a new rehabilitation strategy for balance dysfunction caused by cerebellar infarction.

No changes were observed in the self-perceived intensity of vertigo or nystagmus throughout the VR implementation. This indicates that the mechanisms of vestibular function involved in vertigo and balance are distinct. The intensity of self-perceived vertigo, which directly reflects the asymmetry of the vestibular function similar to head-shaking nystagmus, followed a similar pattern, and comparable results were observed in the patient. Moreover, balance function involves not only vestibular function but also visual and somatosensory inputs that are weighted according to environmental factors and individual capacities to form appropriate sensory responses [64]. In addition, in patients with unilateral peripheral vestibular impairments, there is a low correlation between the intensity of self-perceived vertigo and balance function [65, 66]. Considering these aspects, vertigo is largely a direct reflection of vestibular involvement, whereas balance function reflects not only vestibular function but also the weighting of other sensory inputs. Therefore, in the patient in this case report, the correction of excitability between the left and right vestibular nuclei may have been insufficient, which explains why no changes were observed in the self-perceived intensity of vertigo and nystagmus. However, sensory reweighting compensated for this vestibular excitability insufficiency, suggesting an improvement in balance function. The combination of VR with repetitive transcranial magnetic stimulation in patients with chronic vertigo results in significant improvements compared with VR alone [67]. The potential benefits of combining these neuromodulation devices should be promptly explored. However, notably, in this report, the mCTSIB was limited to measuring the CoP using a force plate, making it difficult to conclusively determine the effects on balance and vestibular functions under each condition.

This study has some limitations. First, this was a single-case study; thus, we cannot rule out the possibility of spontaneous remission or the effects of other rehabilitation interventions. In addition, unlike a single-case design, no baseline period was adopted, and the effects of VR were not compared with those of conventional rehabilitation. Therefore, future studies should use single-case designs or conduct large-scale studies to compare the effects of conventional rehabilitation. Second, the vestibular function tests in this report included the SVV and HIT, which is a screening test for the VOR and VOR cancellation. However, the HIT is insufficiently sensitive to detect mild vestibular impairment [29]. Moreover, these evaluations were conducted by a single assessor, and measurement bias, particularly in subjective assessments, such as HIT and VOR cancellation by physical therapists, should be considered. In particular, the technique and interpretation of the results of VOR cancellation and HIT may be inadequate if the evaluator has limited experience [68, 69]. Moreover, HIT and VOR cancellation are not commonly performed by physical therapists. In this study, notably, the quality of VR was ensured as the physical therapist who conducted the VR was a single practitioner who had received adequate training from experts. Third, evaluating postural control and its changes solely based on the CoP measured using a stabilometer is challenging. Fourth, the Dizziness Handicap Inventory (DHI) could not be implemented for the patient as a self-reported outcome measure for dizziness or vertigo because it contains questions that are not applicable to hospitalized patients. DHI provides a more sensitive and detailed assessment of subjective dizziness or vertigo [70]. Future studies should involve the quantification of VOR and VOR cancellation using video HIT, vestibular evoked myogenic potentials, accelerometers to measure trunk and head motion indices, DHI, and electromyography to assess vestibular function and postural control more sensitively and from multiple perspectives.

## Conclusions

Despite these limitations, this case report suggests that VR may be beneficial for improving balance functions in patients with balance impairment and rotational vertigo due to left cerebellar infarction. Furthermore, improvements were noted in patient-reported outcomes related to instability in ADLs, and a reduction in subjective difficulty experienced in ADLs was observed. However, no changes were observed in the intensity of self-perceived vertigo or nystagmus, and careful consideration is required regarding the effectiveness for these symptoms. Future investigations are required to explore the effects of VR on lesions in the vestibular regions, including the cerebellum, considering the timing of the intervention, suitable cases, and appropriate frequency of sessions in greater detail.

## References

[1] Karabulut M, Van Laer L, Hallemans A (2023). Chronic symptoms in patients with unilateral vestibular hypofunction: systematic review and meta-analysis. Front Neurol.

[2] Geurts AC, de Haart M, van Nes IJ, Duysens J (2005). A review of standing balance recovery from stroke. Gait Posture.

[3] Kwakkel G, Wagenaar RC, Kollen BJ, Lankhorst GJ (1996). Predicting disability in stroke-a critical review of the literature. Age Ageing.

[4] Duracinsky M, Mosnier I, Bouccara D, Sterkers O, Chassany O (2007). Literature review of questionnaires assessing vertigo and dizziness, and their impact on patients' quality of life. Value Health.

[5] Wagner AR, Chaudhari AM, Merfeld DM (2021). Might vestibular “noise” cause subclinical balance impairment and falls?. Int J Phys Med Rehabil.

[6] Kim KJ, Gimmon Y, Millar J, Brewer K, Serrador J, Schubert MC (2021). The instrumented timed “Up & Go” test distinguishes turning characteristics in vestibular hypofunction. Phys Ther.

[7] Agrawal Y, Carey JP, Della Santina CC, Schubert MC, Minor LB (2009). Disorders of balance and vestibular function in US adults: data from the National Health and Nutrition Examination Survey, 2001-2004. Arch Intern Med.

[8] Khan S, Chang R (2013). Anatomy of the vestibular system: a review. NeuroRehabilitation.

[9] Baier B, Thömke F, Wilting J, Heinze C, Geber C, Dieterich M (2012). A pathway in the brainstem for roll-tilt of the subjective visual vertical: evidence from a lesion-behavior mapping study. J Neurosci.

[10] Dieterich M, Bense S, Lutz S, Drzezga A, Stephan T, Bartenstein P, Brandt T (2003). Dominance for vestibular cortical function in the non-dominant hemisphere. Cereb Cortex.

[11] Bense S, Stephan T, Yousry TA, Brandt T, Dieterich M (2001). Multisensory cortical signal increases and decreases during vestibular galvanic stimulation (fMRI). J Neurophysiol.

[12] Arnold SA, Stewart AM, Moor HM, Karl RC, Reneker JC (2017). The effectiveness of vestibular rehabilitation interventions in treating unilateral peripheral vestibular disorders: a systematic review. Physiother Res Int.

[13] Fatima SN, Tanveer F, Shoukat F, Ahmad A, Siddique K (2022). Effects of balance training with and without gaze stabilization exercises on clinical outcomes in elderly patients with chronic dizziness: a randomized controlled trial. J Bodyw Mov Ther.

[14] Tokle G, Mørkved S, Bråthen G (2020). Efficacy of vestibular rehabilitation following acute vestibular neuritis: a randomized controlled trial. Otol Neurotol.

[15] Geraghty AW, Essery R, Kirby S (2017). Internet-based vestibular rehabilitation for older adults with chronic dizziness: a randomized controlled trial in primary care. Ann Fam Med.

[16] Mitsutake T, Sakamoto M, Ueta K, Oka S, Horikawa E (2017). Effects of vestibular rehabilitation on gait performance in poststroke patients: a pilot randomized controlled trial. Int J Rehabil Res.

[17] Meng L, Liang Q, Yuan J (2023). Vestibular rehabilitation therapy on balance and gait in patients after stroke: a systematic review and meta-analysis. BMC Med.

[18] Tramontano M, Bergamini E, Iosa M, Belluscio V, Vannozzi G, Morone G (2018). Vestibular rehabilitation training in patients with subacute stroke: a preliminary randomized controlled trial. NeuroRehabilitation.

[19] Dieterich M, Brandt T (2019). Perception of verticality and vestibular disorders of balance and falls. Front Neurol.

[20] Zwergal A, Feil K, Schniepp R, Strupp M (2020). Cerebellar dizziness and vertigo: etiologies, diagnostic assessment, and treatment. Semin Neurol.

[21] Amari K, Kudo Y, Watanabe K, Yamamoto M, Takahashi K, Tanaka O, Johkura K (2016). Spontaneous, headshaking, and positional nystagmus in post-lateral medullary infarction dizziness. J Neurol Sci.

[22] Vachha BA, Massoud TF, Huang SY (2022). Anatomy of the cerebral cortex, lobes, and cerebellum. Neuroimaging Clin N Am.

[23] Ito M (1998). Cerebellar learning in the vestibulo-ocular reflex. Trends Cogn Sci.

[24] Piscicelli C, Pérennou D (2017). Visual verticality perception after stroke: a systematic review of methodological approaches and suggestions for standardization. Ann Phys Rehabil Med.

[25] Clarke AH, Schönfeld U, Helling K (2003). Unilateral examination of utricle and saccule function. J Vestib Res.

[26] Clarke AH, Schönfeld U, Hamann C, Scherer H (2001). Measuring unilateral otolith function via the otolith-ocular response and the subjective visual vertical. Acta Otolaryngol Suppl.

[27] Dieterich M, Brandt T (1993). Ocular torsion and tilt of subjective visual vertical are sensitive brainstem signs. Ann Neurol.

[28] Kim HA, Hong JH, Lee H (2008). Otolith dysfunction in vestibular neuritis: recovery pattern and a predictor of symptom recovery. Neurology.

[29] Beynon GJ, Jani P, Baguley DM (1998). A clinical evaluation of head impulse testing. Clin Otolaryngol Allied Sci.

[30] Takemori S, Ono M, Maeda T (1979). Cerebral contribution to the visual suppression of vestibular nystagmus. Arch Otolaryngol.

[31] Antoniadou E, Kalivioti X, Stolakis K, Koloniari A, Megas P, Tyllianakis M, Panagiotopoulos E (2020). Reliability and validity of the mCTSIB dynamic platform test to assess balance in a population of older women living in the community. J Musculoskelet Neuronal Interact.

[32] Di Berardino F, Filipponi E, Barozzi S, Giordano G, Alpini D, Cesarani A (2009). The use of rubber foam pads and "sensory ratios" to reduce variability in static posturography assessment. Gait Posture.

[33] Micarelli A, Viziano A, Micarelli B, Di Fulvio G, Alessandrini M (2021). Usefulness of postural sway spectral analysis in the diagnostic route and clinical integration of cervicogenic and vestibular sources of dizziness: a cross-sectional preliminary study. J Vestib Res.

[34] Oppenheim U, Kohen-Raz R, Alex D, Kohen-Raz A, Azarya M (1999). Postural characteristics of diabetic neuropathy. Diabetes Care.

[35] Fujimoto C, Kamogashira T, Kinoshita M (2014). Power spectral analysis of postural sway during foam posturography in patients with peripheral vestibular dysfunction. Otol Neurotol.

[36] Yamamoto T, Suzuki Y, Nomura K (2011). A classification of postural sway patterns during upright stance in healthy adults and patients with Parkinson’s disease. J Adv Comput Intell Intell Inform.

[37] Suzuki Y, Nakamura A, Milosevic M (2020). Postural instability via a loss of intermittent control in elderly and patients with Parkinson's disease: a model-based and data-driven approach. Chaos.

[38] Dieterich M, Brandt T (1992). Wallenberg's syndrome: lateropulsion, cyclorotation, and subjective visual vertical in thirty-six patients. Ann Neurol.

[39] Buckley E, Mazzà C, McNeill A (2018). A systematic review of the gait characteristics associated with cerebellar ataxia. Gait Posture.

[40] Riley DS, Barber MS, Kienle GS (2017). CARE guidelines for case reports: explanation and elaboration document. J Clin Epidemiol.

[41] Balci BD, Akdal G, Yaka E, Angin S (2013). Vestibular rehabilitation in acute central vestibulopathy: a randomized controlled trial. J Vestib Res.

[42] Schubert MC, Migliaccio AA (2019). New advances regarding adaptation of the vestibulo-ocular reflex. J Neurophysiol.

[43] Nelson AB, Krispel CM, Sekirnjak C, du Lac S (2003). Long-lasting increases in intrinsic excitability triggered by inhibition. Neuron.

[44] Zhang S, Liu D, Tian E, Wang J, Guo Z, Kong W (2022). Central vestibular dysfunction: don't forget vestibular rehabilitation. Expert Rev Neurother.

[45] Whyte CA, Petrock AM, Rosenberg M (2010). Occurrence of physiologic gaze-evoked nystagmus at small angles of gaze. Invest Ophthalmol Vis Sci.

[46] Gray VL, Ivanova TD, Garland SJ (2014). Reliability of center of pressure measures within and between sessions in individuals post-stroke and healthy controls. Gait Posture.

[47] Aryan R, Inness E, Patterson KK, Mochizuki G, Mansfield A (2023). Reliability of force plate-based measures of standing balance in the sub-acute stage of post-stroke recovery. Heliyon.

[48] Perry J, Garrett M, Gronley JK, Mulroy SJ (1995). Classification of walking handicap in the stroke population. Stroke.

[49] Bijleveld-Uitman M, van de Port I, Kwakkel G (2013). Is gait speed or walking distance a better predictor for community walking after stroke?. J Rehabil Med.

[50] Franchignoni F, Horak F, Godi M, Nardone A, Giordano A (2010). Using psychometric techniques to improve the Balance Evaluation Systems Test: the mini-BESTest. J Rehabil Med.

[51] Chinsongkram B, Chaikeeree N, Saengsirisuwan V, Viriyatharakij N, Horak FB, Boonsinsukh R (2014). Reliability and validity of the Balance Evaluation Systems Test (BESTest) in people with subacute stroke. Phys Ther.

[52] Van Criekinge T, Heremans C, Burridge J (2024). Standardized measurement of balance and mobility post-stroke: Consensus-based core recommendations from the third Stroke Recovery and Rehabilitation Roundtable. Neurorehabil Neural Repair.

[53] Baker PS, Bodner EV, Allman RM (2003). Measuring life-space mobility in community-dwelling older adults. J Am Geriatr Soc.

[54] Hall CD, Herdman SJ, Whitney SL (2022). Vestibular rehabilitation for peripheral vestibular hypofunction: an updated clinical practice guideline from the Academy of Neurologic Physical Therapy of the American Physical Therapy Association. J Neurol Phys Ther.

[55] Chinsongkram B, Chaikeeree N, Saengsirisuwan V, Horak FB, Boonsinsukh R (2016). Responsiveness of the Balance Evaluation Systems Test (BESTest) in people with subacute stroke. Phys Ther.

[56] Garland SJ, Willems DA, Ivanova TD, Miller KJ (2003). Recovery of standing balance and functional mobility after stroke. Arch Phys Med Rehabil.

[57] Connell LA, Lincoln NB, Radford KA (2008). Somatosensory impairment after stroke: frequency of different deficits and their recovery. Clin Rehabil.

[58] Sato K, Ogawa T (2024). Correlation between trunk function improvement and recovery of activities of daily living after stroke in older adult patients. Neurol Res.

[59] Kim HE, Cho KH (2023). Factor analysis related to the change in activities of daily living performance of stroke patients. Biomed Res Int.

[60] Mahboobin A, Loughlin P, Atkeson C, Redfern M (2009). A mechanism for sensory re-weighting in postural control. Med Biol Eng Comput.

[61] Sprenger A, Wojak JF, Jandl NM, Helmchen C (2017). Postural control in bilateral vestibular failure: its relation to visual, proprioceptive, vestibular, and cognitive input. Front Neurol.

[62] Barra J, Marquer A, Joassin R, Reymond C, Metge L, Chauvineau V, Pérennou D (2010). Humans use internal models to construct and update a sense of verticality. Brain.

[63] Saeys W, Herssens N, Verwulgen S, Truijen S (2018). Sensory information and the perception of verticality in post-stroke patients. Another point of view in sensory reweighting strategies. PLoS One.

[64] Horak FB (2006). Postural orientation and equilibrium: what do we need to know about neural control of balance to prevent falls?. Age Ageing.

[65] Kammerlind AS, Larsson BP, Ledin T, Skargren E (2005). Reliability of clinical balance tests and subjective ratings in dizziness and disequilibrium. Adv Physiother.

[66] Nam GS, Jung CM, Kim JH, Son EJ (2018). Relationship of vertigo and postural instability in patients with vestibular schwannoma. Clin Exp Otorhinolaryngol.

[67] Koganemaru S, Goto F, Arai M (2017). Effects of vestibular rehabilitation combined with transcranial cerebellar direct current stimulation in patients with chronic dizziness: an exploratory study. Brain Stimul.

[68] Lelli DA, Tse D, Vaccani JP (2019). Competence of final year otolaryngology residents with the bedside head impulse test. J Otolaryngol Head Neck Surg.

[69] Lelli DA, Rourke R, Tse D (2024). Competence of senior otolaryngology residents with the bedside head impulse test-has there been improvement after 5 years of competency by design?. J Otolaryngol Head Neck Surg.

[70] Zamyslowska-Szmytke E, Politanski P, Jozefowicz-Korczynska M (2021). Dizziness Handicap Inventory in clinical evaluation of dizzy patients. Int J Environ Res Public Health.

